# Treating Hypotension in Preterm Neonates With Vasoactive Medications

**DOI:** 10.3389/fped.2018.00086

**Published:** 2018-04-13

**Authors:** Chloe Joynt, Po-Yin Cheung

**Affiliations:** ^1^Department of Pediatrics, University of Alberta, Edmonton, AB, Canada; ^2^Department of Pharmacology and Surgery, University of Alberta, Edmonton, AB, Canada; ^3^Centre for the Studies of Asphyxia and Resuscitation, Edmonton, AB, Canada

**Keywords:** newborn, prematurity, inotropes, catecholamines, blood pressure, hypotension

## Abstract

Preterm neonates often have hypotension which may be due to various etiologies. While it is controversial to define hypotension in preterm neonates, various vasoactive medications are commonly used to provide the cardiovascular support to improve the blood pressure, cardiac output, or to treat shock. However, the literature on the systemic and regional hemodynamic effects of these antihypotensive medications in neonates is deficient and incomplete, and cautious translation of findings from other clinical populations and animal studies is required. Based on a literature search on published reports, meta-analytic reviews, and selected abstracts, this review discusses the current available information on pharmacologic actions, clinical effects, and side effects of commonly used antihypotensive medications including dopamine, dobutamine, epinephrine, norepinephrine, vasopressin, and milrinone in preterm neonates.

Antihypotensive treatments are often started in response to a low blood pressure (BP) or signs of low cardiac output (CO) in critically ill neonates. The challenge for clinicians in the neonatal intensive care unit (NICU) is to dissect out the etiology of the hemodynamic changes, decide if the changes are pathologic or transitionally appropriate, and then tailor the treatment regimen for the patient, the condition, and gestation. This process all occurs while being cognizant that a hemodynamic state evolves throughout the chronological age of the neonate and the course of illness and is affected by concurrent treatments, such as ventilation. Studies in Europe, North America, and Australia all highlight that practices are variable across countries and continents with respect to which patient, when and how to treat with cardiotonic drugs (1–6).

A national Canadian database reported that 10% of neonates of <29 weeks had been treated with inotropes on days 1–3 (0–36% within the 27 NICUs) (1). The treated neonates were less likely to have received antenatal corticosteroids, more likely have a smaller birthweight, a higher SNAPS II, TRIPS score, and need for ventilation, and had a higher mortality and incidence of intraventricular hemorrhage. Recently, a Norwegian population database study indicated that 2.7% of all NICU patients received inotropes at any point of their NICU stay; 28 and 4.1% of <28 and <36 weeks of gestation, respectively, and 13% of <1,500 g infants (2). These numbers are similar to those reported by Lasky et al. in American NICU (7). Multiple inotropes were associated with an increased mortality. Indeed, the use of inotropes was associated with an increased mortality, after adjusting for gender, gestation, and 5-min Apgar.

## Defining Hypotension

Given variations in gestation, birthweight, and perinatal states along with the cardiopulmonary transition, it is challenging to provide a robust definition of hypotension. Many practitioners still define hypotension as a mean BP lower than the gestational age of the baby, most likely deferring to its ease of use (5). However, BP increases over time after birth, and it is cautious to use this definition past the first days of life. Although it has not been validated in larger studies, BP nomograms may be a promising alternative to help define hypotension, especially for those patients with extremely low BP.

Transitional and neonatal physiology including fetal shunts as well as disease pathology and iatrogenic effects of concurrent treatments can all contribute to the disruption of hemodynamic homeostasis. During the postnatal transition after birth, there are significant changes in the output of both ventricles as well as systemic and pulmonary vascular resistance which contribute to the initial “physiological” decrease in BP. However, little information is available regarding the minimal effective BP and or blood flow for tissue perfusion in this postnatal hemodynamic state. Permissive hypotension becomes a managerial approach when “hypotension” or low BP is noted in neonates, especially the preterm neonates. It is contemporary to adopt a functional definition of hypotension in the context of clinical effects of hypotension using clinico-biochemical markers of tissue hypo-perfusion.

In pathological conditions such as hypoxia and sepsis, poor myocardial contractility and pulmonary hypertension, which can further be aggravated by acidosis, cause hypotension and tissue hypo-perfusion (8). Systemic vasodilation or vasoplegia is often found in sepsis, profound hypoxia, or postoperatively. Sick neonates often have impaired autoregulation or a redistribution of organ blood flow that alters the relationship between BP, CO, and organ perfusion (9, 10).

## Treating Hypotension or Not

A systematic review comparing permissive and BP value-based hypotension therapeutic strategies in the very preterm infant highlighted the paucity of quality data for either therapeutic approach and little published evidence to link hypotension or treatment to short- and long-term outcomes (11). Faust et al. reported that in neonates born at <32 weeks and <1,500 g, inotropic support was associated with intraventricular hemorrhage and hypotension in the first 24 h and had an increase in mortality (12). Batton et al. further reported motor, language, and cognitive delay at 18–22 months follow-up in a cohort of hypotensive neonates <27 weeks who received BP treatments (4). Interestingly, EPIPAGE 2 cohort study for neonates born at <29 weeks recently reported that those who received antihypotensive treatment for isolated hypotension in the first 72 h had a higher survival rate without major morbidity than matched infants without hypotension treatment (13).

Given the paucity of randomized trials of antihypotensive therapeutics and contradictory data from large cohort studies, there is wide variation in therapies using protocolized or clinical, laboratory and/or technology-based approach (6, 14). While it still remains common to treat hypotension with a fluid boluses followed by an infusion of antihypotensive medication, fluid bolus administration may not be beneficial and indeed be harmful (6, 15). Conversely, in neonates with compromised intravascular volume due to blood or fluid loss, or capillary leak, fluid administration is essential for proper hemodynamic responses and augments effects of inotropes or pressors. Therefore, fluid therapy should be given with meticulous attention to the intravascular fluid balance or measures, especially for those extremely preterm neonates (16).

## Vasoactive Agents

Burns et al. reported that dopamine was the most commonly used vasoactive agent with a median duration of administration of 46 h and a median maximum dose of 10 µg/kg/min, followed by epinephrine (33 h and 0.3 µg/kg/min, respectively) and dobutamine (22 h and 8.3 µg/kg/min, respectively), with the increasing use of milrinone, norepinephrine, and vasopressin (2). Dopamine, dobutamine, epinephrine, and norepinephrine are categorized as catecholamines as their chemical structure contains a catechol and an amine group. These catecholamines stimulate adrenergic (and/or dopaminergic and serotonergic) receptors not only in the sympathetic nervous system at the synapse but also in the cardiomyocytes, vascular smooth muscle cells, and other extravascular parenchymal cells resulting in complex cardiovascular, renal, and endocrine actions (17). However, the expression in cardiovascular adrenoreceptors can be altered by critical illness, extended catecholamine use, relative adrenal insufficiency as well as gestational age and maturity (18). In the failing myocardium, there is a downregulation of β-1 adrenoreceptors, an uncoupling of β-2 adrenoreceptors from adenylyl cyclase, and a decreased cAMP production (19).

## Dopamine

Dopamine is an endogenous catecholamine precursor of norepinephrine with sympathetic and neuroendocrine properties (Table 1). It directly stimulates dopaminergic and adrenergic receptors or indirectly, usually in the inotropic dose range, stimulates dopamine-2 receptor to release norepinephrine stored in the peripheral sympathetic nerve endings in the myocardium (17, 20–23). The latter indirect mechanism renders dopamine a poor choice for long-term inotropic therapy due to the eventual depletion of endogenous stores.

**Table 1:** Summary of antihypotensive medications in preterm neonates.

Medication	Suggested dosing^a^	Predominant mechanism	Potential clinical uses	Potential side effects
Dopamine	≤ 2 µg/kg/min	Dopaminergic receptors agonism—*proposed* renotubular effects, intestinal and coronary vasodilation.	A vasopressor which increases blood pressure if the “hypotension” requires treatment.	Causes tachycardia. Aggravates stress to both ventricles due to increased afterload. At ≥10 µg/kg/min, may cause right to left ductal shunt. Decreased effect in long term as indirect pathway substrate becomes depleted.
	≥5–10 µg/kg/min	β-1Adrenergic receptors agonism—mainly chronotropy	– Increases urine output (increased renal perfusion pressure and/or natriuresis).	
	≥10 µg/kg/min	α-1Adrenergic receptors agonism—vasoconstriction	– Has a modest effect in cardiac output.	

Dobutamine	≥3–15 µg/kg/min	β-1Adrenergic receptors agonism—chronotropy and inotropy	An inotrope which increases cardiac output without vasoconstriction, e.g., cardiogenic shock.	Causes tachycardia. Avoid in cardiac outflow tract obstructions, e.g., infants of diabetic mothers.
		β-2 Adrenergic receptors agonism—peripheral vasodilation	– Has unpredictable effect on blood pressure.	

Epinephrine^b^	≥0.02–0.1 µg/kg/min	β-1and some β-2 Adrenergic receptors agonism—chronotropy and inotropy, with modest decrease in PVR	An inotrope with vasopressive action, e.g., hypotension with decreased cardiac contractile function with or without vasoplegia, e.g., septic shock, asphyxia.	Causes hyperlactatemia and hyperglycemia. Causes tachycardia. May increase myocardial oxidative stress. Use with caution in cardiac outflow tract obstructions, e.g., infants of diabetic mothers.
	≥0.1 µg/kg/min	α-1Adrenergic receptors agonism—vasoconstriction.	– At 0.02–0.05 µg/kg/min, may increase cardiac output more than SVR.	

Norepinephrine^b^	0.02–0.4 µg/kg/min	α-1 (> β-1 > β-2) Adrenergic receptors agonism—potent vasoconstriction (and mild inotropy)	A vasopressor which serves as an adjunct to other catecholamines at low dose. – Conditions with significant vasoplegia in refractory sepsis, post-surgical inflammation, asphyxia. – May have mild pulmonary vasodilation effect.	Tachycardia. May affect regional tissue perfusion due to potent vasoconstriction.

Vasopressin	0.0002–0.005 U/kg/min	V_1a (>V2)_ receptors agonism—vasoconstriction.	A vasopressor which increases blood pressure in catecholamine-resistant hypotension or shock	Hyponatremia. Transient thrombocytopenia. Liver necrosis. Limb necrosis.

Milrinone	0.25–0.75 µg/kg/min	Phosphodiesterase type III inhibition with increased cAMP levels—inotropy, lusitropy, and pulmonary (and possible systemic) vasodilation.	– An inotrope/luisitrope with mild pulmonary vasodilation (*inodilator*), e.g., pulmonary hypertension with ventricular dysfunction, post-PDA ligation. – Loading do e is not needed while the onset of action may take 1–2 h.	Slow onset. Tachycardia. Hypotension if decreased intravascular volume or administration of a loading dose. Decreased platelet aggregation. Decreased clearance with kidney dysfunction.

*PVR, pulmonary vascular resistance; SVR, systemic vascular resistance*.

*^a^There are variations related to birthweight, gestational age, and disease severity with overlaps in dose–responses of specific receptors agonism. At high doses, antihypotensive medications should be used with caution due to risks for adverse effects, in addition to non-selective receptors stimulation*.

*^b^Use with caution at >0.2 μg/kg/min. Indeed, 0.2 µg/kg/min of epinephrine equates to approximately an hourly administration of bolus epinephrine (10 µg/kg) during resuscitation*.

Our evidence in preterm neonates is mostly inferred from animal studies and small clinical trials (24–28). The effects of dopamine at different doses, different gestations, and chronological ages often receive discussion (11). In a preterm population, the *dopaminergic* receptors expressed in renal, mesenteric, and coronary vascular beds *may* be activated at very small dosages (0.5–2 µg/kg/min), in addition to those regulating the tubular ion fluxes (29). Clinical changes in urine output may be dopaminergic in nature or due to increased renal blood flow and perfusion pressure secondary to its cardiovascular effects. In addition, Seri also suggested that the α-1 *vasopressive* effects occur at 2–5 µg/kg/min and overlap with the β-1 *inotropic* effects which may be at 4–10 µg/kg/min in premature infants (29). In a study of 18 premature infants, a high dose dopamine (>10 µg/kg/min) had an unpredictable effect on pulmonary arterial pressures and changed the systemic to pulmonary arterial pressures ratio allowing right to left shunting across the ductus arteriosus and potentially aggravating tissue hypoxia (24, 26, 30). Furthermore, the clearance of dopamine is gestation-dependent and can be markedly decreased in sick neonates (31, 32). Dose–responses also vary in neonates with different underlying pathophysiologies, requiring the titration of dosage based on patient effect and not standardized dosing (33).

There are multiple recent reviews or meta-analyses of dopamine use as an antihypotensive agent for premature neonates (15, 34–36). A Cochrane review indicated that dopamine has a modest increase in mean or systolic BP, especially in comparison to dobutamine (NNT = 4.4, 95% CI 2.9–7.7) (36, 37). Despite this vasopressive, but not inotropic, effect, there is no difference in short- or long-term morbidities between dopamine- and dobutamine-treated neonates (36). It is controversial regarding the potential effects of dopamine on cerebral autoregulation (38). Of note, high doses may place the ventricle under stress and, as demonstrated in animal models, may potentiate right to left shunting (37, 39). Nonetheless, its ubiquitous nature in many hospital pharmacies, quick onset of action, and historical familiarity continue to play a role in the dopamine use in NICU.

## Dobutamine

Dobutamine is a synthetic catecholamine that acts predominantly on β-1 adrenoreceptors to increase cardiac contractility (Table 1). It may cause tachycardia, thus increasing myocardial oxygen consumption, at higher doses with the potential for mild peripheral vasodilation (40). Dobutamine, however, may have a role in the transition of preterm neonates who have a low CO and an increased systemic vascular resistance such as polycythemia, asphyxia, or severe acidosis.

Dobutamine, when compared to dopamine, may have better CO or SVC flows in hypotensive preterm neonates (37, 41, 42). Interestingly, dobutamine (9.1 ± 1.1 µg/kg/min) improved stroke volume and CO of preterm neonates within 20 min, whereas increases in flow velocities at the cerebral, mesenteric, and renal arteries were observed 8–10 h later (43). No difference was found in mortality, intraventricular or periventricular hemorrhage, morbidities at term-corrected age, and outcomes at early childhood (37, 43, 44).

Perhaps, given the comparative studies of dopamine and dobutamine which show that dopamine increases the readily accessible measurement of BP, while dobutamine may increase CO or SVC flows, a measurement often requiring echocardiography, a recent study in the trend of antihypotensive use indicates that dobutamine has decreased from second to fourth most commonly used medication in the treatment of neonatal hypotension (45).

## Epinephrine

Epinephrine is an endogenous catecholamine that acts directly and dose-dependently on α-1 (>0.1 µg/kg/min) and α-2, β-1 and β-2 (0.02–0.1 µg/kg/min) adrenoreceptors, with vasopressive and inotropic actions, respectively (Table 1). There may be a modest decrease in pulmonary vascular resistance as well as vasodilation of renal and mesenteric vasculature at low doses. As doses escalate, vasoconstriction can become intense, tachycardia is pronounced, blood flow to the gut and kidneys decreases, and increased oxygen consumption occurs, although there is still some inotropic action and blood flow is increased to the brain and heart (29). This may put additional stress on an already compromised neonatal heart and peripheral tissues. The pediatric vasoactive inotropic score equates 0.1 µg/kg/min of epinephrine to 10 µg/kg/min of dopamine (46). Pharmacodynamic studies in a handful of term and preterm neonates post cardiac surgery demonstrated that a lower birthweight is associated with a lower epinephrine clearance and amplitude in the change of heart rate and BP. It is postulated that this is due to the relative immaturity of the neonatal myocardium, which precludes a significant increase in stroke volume and a variation in β-1 and β-2 adrenergic receptor density related to age.

Epinephrine at >0.5 µg/kg/min causes excessive vasoconstriction and possibly disorganized energy utilization leading to a decreased CO. In addition to hyperglycemia, hyperlactatemia is a known sequelae of epinephrine infusions due to the stimulation of glycogenolysis *via* the activation of β-2 adrenergic receptors and anaerobic metabolism with α-adrenergic vasoconstriction (47). Furthermore, sarcolemmal rupture and increased cytoplasmic calcium deposits with resulting decreased myocardial compliance were found with prolonged epinephrine infusions in neonatal piglets (48).

Human neonatal literature on epinephrine is scarce and mostly in preterm neonates (49–52). Pellicer et al. compared the effect of dopamine (2.5–10 µg/kg/min) and epinephrine (0.125–0.5 µg/kg/min) on cerebral hemodynamics measured by near infrared spectroscopy (51). Epinephrine has similar increases in BP, but more in heart rate at the highest dose range, when compared with dopamine. Cerebral blood flow increased significantly in the more preterm neonates (<28 weeks) with epinephrine and in the more mature neonates (28–32 weeks) with dopamine. This study, however, does not provide information regarding α- and β-adrenergic agonism. Given the limited literature, there are insufficient data to make recommendations for the use of epinephrine in the preterm neonatal population (53).

## Norepinephrine

Norepinephrine is an endogenous sympathomimetic amine that acts on the vascular and myocardial α-1 receptors with a mild to moderate β-1 adrenoreceptor agonism (Table 1). As the effect on β-2 adrenoreceptors is minimal, norepinephrine has combined inotropic and peripheral vasoconstrictive effects (54). The clinical literature on norepinephrine use in neonates is predominantly involving refractory shock and demonstrates increased BP, improved oxygenation, and decreased serum lactate within hours of initiation (55). While improved systemic BP may contribute to the improved oxygenation, norepinephrine may also cause pulmonary vasodilation, particularly in neonates with preexisting increases in basal pulmonary vascular tone, as can be found in severe sepsis (56–58).

A pediatric norepinephrine pharmacokinetic and pharmacodynamics study (0.5–3 µg/kg/min) included 11 neonates and indicated that variabilities were related to weight, age, and severity of illness which was related to the production and clearance of norepinephrine (59). We found two retrospective studies of norepinephrine use in preterm neonates (*n* = 48, <32 weeks and *n* = 30, <34 weeks) (60, 61). Both studies demonstrated improvements in BP and oxygenation parameters within 3–8 h, with variable effect on urine output. Of note, two-thirds of the patients had sepsis with the majority receiving norepinephrine as an adjunctive therapy. In the studies, tachycardia was very common and mortality was high (30 and 46%, respectively).

Norepinephrine is often used as a second- or a third-line antihypotensive agent. Adapting practices from pediatric and pediatric cardiovascular intensivists, in the presence of severe vasoplegia or right ventricular failure, the addition of norepinephrine 0.02–0.05 µg/kg/min when epinephrine doses are reaching >0.1–0.2 µg/kg/min allows for the de-escalation of epinephrine and its concurrent side effects of lactic academia and hyperglycemia (personal observations). Further, norepinephrine may improve cardiac performance of both ventricles and coronary systolic BP to assist the right ventricle, especially during ventricular stress (62). However, if the ventricles contractility is impaired, caution should be exercised with excessive systemic vasoconstriction which results in increased afterload and myocardial oxygen demand and worsens unsupported ventricular function.

## Vasopressin

In the last decade, there was a growing use of vasopressin in neonates for various conditions including sepsis, catecholamine and corticosteroid-resistant shock, hypotension from ventricular outflow tract obstructions, post cardiopulmonary bypass for congenital cardiac surgery, and pulmonary hypertension (63–67). Vasopressin, or arginine vasopressin (AVP), is an endogenous substance with three subtypes that mediates vasoconstriction (V_1_), regulates water reabsorption (V_2_), and has effects on the central nervous system including the release of adrenocorticotrophic hormone (V_3_) (68, 69). The vascular effects of vasopressin occur *via* the V_1a_ and V_2_ receptors in the cardiovascular system. The V_1a_ and V_2_ receptors induce vasoconstriction and vasodilation, respectively, with the potent V1 vasoconstrictive effects predominating when it is used as an exogenous infusion (Table 1) (70). Experimental studies indicate that low-dose AVP causes selective vasodilation in pulmonary, coronary, and cerebral vasculature under hypoxic conditions while causing vasoconstriction in other vascular beds (71–74).

The neonatal use of vasopressin has been predominantly for catecholamine-resistant shock, hypothetically tackling the hypotension *via* the depletion of endogenous AVP in a critically ill state as well as the vasoplegia unresponsive to catecholamines (75, 76). An increase in the mean BP and the ability to decrease inotrope score were not accompanied by an improved survival and an increased end-organ perfusion (77). This is not surprising in this rescue use of vasopressin in the late shock process. A randomized blinded pilot study compared dopamine (5–20 µg/kg/min) and vasopressin (0.0002–0.0007 µg/kg/min) as an initial antihypotensive agent and found no difference in the efficacy and time to response in the treatment of 20 neonates of <30 weeks gestation with hypotension at <24 h (78). Interestingly, the vasopressin-treated group had lower PaCO_2_ values and less surfactant needed, compared to the dopamine-treated group.

Adverse effects of vasopressin including hyponatremia, transient thrombocytopenia, and liver necrosis have been reported in neonates, with limb necrosis predominantly in terlipressin use (63, 67, 79, 80). Ni et al. recently reported that the vasopressin use (maximum dose of 0.03 ± 0.016 units/kg/h) was not associated with hyponatremia in 21 preterm neonates (340–1,390 g, 23–32 weeks of gestation) (81). The summative literature acknowledges that there was insufficient evidence to recommend or refute the use of AVP due to a lack of high-quality trials.

## Milrinone

Milrinone is a bipyridine derivative that is a phosphodiesterase type III inhibitor that increases intracellular cAMP and calcium concentration through the inhibition of cAMP degradation (Table 1) (82). Milrinone increases myocardial contractility through a cyclic AMP-mediated increase in trans-sarcolemmal calcium flux (inotropy). Milronone also allows calcium resequestration into the sarcoplasma reticulum (vasodilation), and improved actin–myosin dissociation during diastole (lusitropy). Possibly due to its site of action distal to the β adrenoreceptor, the myocardial contractile response of milrinone seems to be preserved with ongoing use (51).

Milrinone may improve left ventricular function and reduce pulmonary (venous and arterial) hypertension as it increases cAMP levels in both cardiomyocytes and pulmonary arterial smooth muscle cells (83). Milrinone has been used in neonates predominantly for pulmonary hypertension and low CO both before and after cardiac surgery but also in preterm neonates at risk for low systemic blood flow (84–91). A randomized controlled trial of milrinone in preterm neonates showed no clear benefit to prevent low SVC flow in the first few days of life (92). A handful of retrospective and case studies of prophylactic milrinone administration to prevent cardiovascular dysfunction after patent ductus arteriosus ligation demonstrated that milrinone might reduce hemodynamic instability during the first 24 h postoperatively (93, 94). However, a recent retrospective comparative study did not confirm a significant cardiovascular or long-term clinical benefit (95).

Animal data have questioned the maturity of phosphodiesterase-3 receptors at birth and the utility of milrinone in the first days of life, but recent data have shown significant changes of CO with milrinone use as early as 12 h of age (88, 96). Milrinone has a half-life of approximately 4 h in neonates, thus rendering a slow onset of action (97, 98). Giaccone et al. recently found that renal clearance was increased in relation to gestational and chronological age in term neonates (up to 10 days) (99). Therefore, the dosage of milrinone needs to be adjusted in renal impairment, prematurity, or early chronologic age to avoid drug accumulation and thus side effects including thrombocytopenia (90, 91, 99).

The multifactorial etiologies of hypotension in preterm neonates require a tailored approach to medical cardiovascular support that focus on more than just maintenance of an acceptable BP. Using of targeted neonatal echocardiography, near infrared spectroscopy, and other hemodynamic/perfusion-monitoring technologies may provide important information regarding the responses to antihypotensive medications, in addition to guiding their use in different scenarios (cardiogenic vs vascular) of hypotension (100, 101). An increase in BP should not be deemed a treatment success if excessive vasoconstriction is causing further stresses to cardiac workload or organ tissue oxygenation (102). Although there are many attempts to demonstrate that treating hypotension, as opposed to low blood flow or decreased CO, will improve mortality and morbidity, the literature is still not yet conclusive. Despite ongoing clinical use, literature is sparse with very few comparative studies, on the neonatal use of dobutamine, epinephrine, norepinephrine, vasopressin or milrinone or other cardiovascular supportive therapies for hypotension in preterm neonates.

## Author Contributions

CJ: conception of the topic, writing the first draft and final edit and approval of the manuscript. P-YC: conception of the topic, editing, and approval of the manuscript.

## Conflict of Interest Statement

The authors declare that the research was conducted in the absence of any commercial or financial relationships that could be construed as a potential conflict of interest.
